# Generation of a Highly Biomimetic Organoid, Including Vasculature, Resembling the Native Immature Testis Tissue

**DOI:** 10.3390/cells10071696

**Published:** 2021-07-05

**Authors:** Tat-Chuan Cham, Fahar Ibtisham, Mohammad Amin Fayaz, Ali Honaramooz

**Affiliations:** Department of Veterinary Biomedical Sciences, Western College of Veterinary Medicine, University of Saskatchewan, Saskatoon, SK S7N 5B4, Canada; tc.cham@usask.ca (T.-C.C.); fmi065@mail.usask.ca (F.I.); ma.fayaz@usask.ca (M.A.F.)

**Keywords:** testis organoid, de novo testis organogenesis, tubulogenesis, testis cell self-assembly

## Abstract

The creation of a testis organoid (artificial testis tissue) with sufficient resemblance to the complex form and function of the innate testis remains challenging, especially using non-rodent donor cells. Here, we report the generation of an organoid culture system with striking biomimicry of the native immature testis tissue, including vasculature. Using piglet testis cells as starting material, we optimized conditions for the formation of cell spheroids, followed by long-term culture in an air–liquid interface system. Both fresh and frozen-thawed cells were fully capable of self-reassembly into stable testis organoids consisting of tubular and interstitial compartments, with all major cell types and structural details expected in normal testis tissue. Surprisingly, our organoids also developed vascular structures; a phenomenon that has not been reported in any other culture system. In addition, germ cells do not decline over time, and Leydig cells release testosterone, hence providing a robust, tunable system for diverse basic and applied applications.

## 1. Introduction

The establishment of an in vitro testis organoid system from dissociated testis cells has become a hot topic for research in regenerative medicine and reproductive biotechnology [1,2,3]. Testis organoids have the potential to be used as an important experimental model for biological investigations or pharmaco-toxicology testing of various factors for their effects on testis development and function [4,5]. The testis is a structurally complex organ with dual roles in the production of male hormones and gametes. Testicular organogenesis occurs during embryonic/fetal stages and as a result of a complex folding of the germinal layers as well as the formation, migration, and assembly of both germ cells and somatic cells [6]. These events lead to compartmentalization of the tissue into two morphologically and functionally distinct tubular and interstitial components. The tubular compartment is comprised of immature testis cords in neonatal males and mature seminiferous tubules (containing a lumen) in adults [7,8,9]. The tubular compartment is constructed primarily by two somatic cell types, peritubular myoid cells (PTMCs) and Sertoli cells, which harbor germ cells of various types undergoing development [10]. Both Sertoli cells and PTMCs release components to construct a basement membrane, separating the tubular and interstitial compartments [11,12]. The interstitial compartment is made of connective tissue, blood vessels, and Leydig or interstitial cells with androgen producing capabilities. Hence, testis organogenesis is viewed as a critical process in the development of male embryos with long-lasting consequences for their reproductive potential. Disorders of the normal testis development and interferences in the production/action of androgens do occur [13], and can predispose the individual to early or long-term complications such as cryptorchid testis, testicular germ cell tumors, or impaired spermatogenesis [14].

The range of in situ studies and manipulations that can be performed during the early embryonic development of testis tissue is extremely limited. A suitable testis organoid system can provide an accessible in vitro model for investigations into the effects of potential factors that can adversely affect testicular morphogenesis. The goal of an ideal testis organoid system is to reconstruct an artificial tissue that is structurally and functionally similar to intact testis tissue. Three criteria have been proposed by Edmonds and Woodruff (2020) to evaluate the testis organoid formation in a given culture system [3,15]. These criteria include (a) testis cell reassembly, (b) the presence of a compartmentalized architecture, and (c) the inclusion of major testis cell types (Sertoli, Leydig, germ, and PTMCs). In particular, testis cells first reassemble into newly formed multicellular structures such as cell aggregates or testis cord-like/tubular structures. The tubulogenic ability of testis cells is described as their capacity to self-assemble into testis cord-like/tubular structures which can contain different cell types and cellular orientations; the final results define their architectural relevance to native testis tissue. Therefore, the degree of de novo testis organogenesis is considered to be higher if more criteria are completed by a given testis organoid culture system.

Since the late 20th century, a wide range of testis organoid culture systems have been introduced to recapitulate de novo testis organogenesis [2,3,16]. Despite tremendous efforts, limitations including the lack of non-rodent models, progressive decrease in the number of germ cells, missing key testis cell types, improper cellular orientation, and incomplete de novo testis organogenesis were reported in these earlier culture systems [15,16,17,18,19,20,21]. Therefore, intense research in the field continues to introduce a suitable testis organoid system that can replicate the in situ form and function of the testis tissue using non-rodent models. Since pigs share physiological and anatomical similarities with humans, they are increasingly viewed as an alternative non-rodent model species for biomedical, pharmaceutical, and xenotransplantation studies [22,23,24]. The availability of neonatal testis tissue after routine castration of piglets provides a consistent supply of source cells to carry out various experiments. Due to these reasons, neonatal porcine testis cells have been selected as the cell sources in the present study for the establishment of testis organoid culture systems.

Testis organoids have tremendous potential to be used in basic and clinical research into etiology of infertility, strategies to preserve fertility potential of immature individuals, and restoration of male fertility [23,25,26]. Knocking-out specific genes such as glial cell line-derived neurotrophic factor (*Gdnf*) is lethal to neonates [27]; hence, testis organoids can also be utilized in studying the effects of neonatal lethal knockouts or mutants on in vitro fate of testis cells. Additionally, testis organoids generated from oncogenic or transgenic testis cells can also provide an important tool with which to study the transformed signaling pathways, genetic modification of carcinogenic cells, and the efficiency of cancer therapy, all which are otherwise difficult to study using other models. A testis organoid system was even used in the pathological assessment of Zika virus infection [28], and hence may also prove valuable in examining the pathogenesis mechanisms of the impaired testis function observed in COVID-19 patients [5,29].

## 2. Materials and Methods

### 2.1. Experimental Design

**Figure 1:**
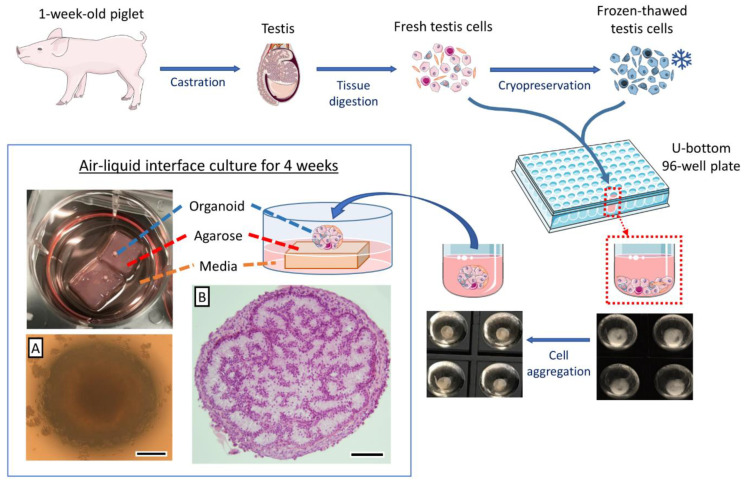
Schematic summary of the procedures for formation of testis organoids. Testis tissue was collected from 1-week-old piglets and digested to isolate testis cells. Testis cells were either used fresh for organoid formation or cryopreserved for later use. The fresh or frozen-thawed testis cells (1.0 × 10^6^ cells/well) were then cultured in U-bottom 96-well plates for 24 h to form cell spheroids. The cell spheroids were subsequently cultured on small blocks (1 × 1 × 0.5 cm) of 1.5% agarose gel base immersed in media. The cell spheroids were further cultured for 4 weeks in this air–liquid interface culture system to form testis organoids, and the media were changed every other day. (**A**) Stereomicroscopic and (**B**) histological micrographs of testis organoids. Scale bars: 300 µm (**A**); 100 µm (**B**).

Figure 1 shows a schematic overview of the procedures leading to the formation of testis organoids. Testes collected at routine castration of 1-week old piglets were enzymatically digested to isolate heterogeneous populations of testis cells. The cells were allowed to form cell spheroids which were then transferred onto agarose gel blocks soaked in the media to be cultured to form testis organoids. The effects of cell density, media supplementation, cell cryopreservation, and hormone stimulation on the formation and characteristics of the testis organoids were also assessed.

### 2.2. Testis Collection and Preparation

Testes were collected through aseptic castration of 1-week-old Yorkshire-cross piglets at our University’s swine center. The testes were kept in Dulbecco’s phosphate-buffered saline (DPBS; Cat. No. 20-031-CV; Mediatech, Manassas, VA, USA), containing 1% antibiotics solution (penicillin and streptomycin; Cat. No. 30-002-CI, Mediatech), and were transported on ice. In the laboratory, the testes were rinsed three times with DPBS prior to processing. The tunica albuginea and excess connective tissue containing collagen fibers, fibroblasts, and fibrocytes, were first removed from the testis tissues. The remaining tissue was mainly testis parenchyma that contained testis tubular and interstitial components, which was further processed for testis cell isolation. Testes were also collected from 1- and 4-week-old piglets to be used as reference tissue for histological assessments.

### 2.3. Testis Cell Isolation

Testis cell isolation was performed using methods previously established in our laboratory (including a three-step enzymatic digestion) [30,31]. Briefly, three or four pairs of testes were used for each enzymatic digestion. Testis parenchyma was separated from the tunica albuginea and thoroughly minced with fine scissors for 5 min, suspended in 15 mL of DPBS, vortexed for 30 s in a test tube shaker (Reax Top; Cat. No. 541-10000; Heidolph Instrument, Schwabach, Germany) and digested with 5 mL of 0.2% collagenase IV (Cat. No. C-153; Sigma-Aldrich, Oakville, Canada), 0.1% hyaluronidase (Cat. No. H-3884; Sigma-Aldrich), and 0.01% DNase (Cat. No. DN25; Sigma-Aldrich) in Dulbecco’s Modified Eagle Medium (DMEM; Cat. No. 10-013-CM; Mediatech) supplemented with 1% *w/v* antibiotics (Cat. No. 30-002-CI; Corning, Manassas, VA, USA) at 37 °C for 15 min. Fetal bovine serum (FBS; Cat. No. A15-701; PAA Laboratories, Toronto, Canada) was added to stop the digestion. To remove cell clumps and undigested tissue segments, the suspension was vortexed for another 30 s and filtered through a 40 µm filter (Cat. No. 3522340; BD Biosciences, San Jose, CA, USA). The filtrate cell suspension was then centrifuged at 500× *g* at 16 °C for 5 min to form a cell pellet and the supernatant was removed. The cell pellet was then re-suspended with 20 mL of the lysis buffer and placed at room temperature (RT) for 30 min for erythrolysis. The lysis buffer contains 156 mmol/L ammonium chloride (NH_4_Cl; Cat. No. A9434; Sigma-Aldrich), 10 mmol/L potassium bicarbonate (KHCO_3_; Cat. No. 237205; Sigma-Aldrich), and 0.1 mmol/L disodium ethylenediaminetetraacetate (Na_2_EDTA; Cat. No. E6635; Sigma-Aldrich) in sterile distilled water. The cell-lysis buffer mixture was then centrifuged at 500× *g* at 16 °C for 5 min and the supernatant was removed. Lastly, the cell pellet was washed with DPBS and re-suspended in 5 mL of DMEM supplemented with 10% knockout serum replacement (KSR, Cat. No. 10828028; Gibco, Thermo Fisher Scientific, Carlsbad, CA, USA). The cell number and viability of the resultant cell suspension was assessed using the trypan blue exclusion assay.

### 2.4. Testis Cell Cryopreservation

The resultant testis cells were either used fresh for organoid formation or cryopreserved using our previously published protocol [32], with minor modifications. Briefly, the cryoprotectant was first made with FBS, DMEM, and dimethyl sulfoxide (DMSO; Cat. No. D2650; Sigma-Aldrich) at a 1:3:1 ratio and stored at 4 °C. The cell suspension was then mixed with ice-cold cryoprotectant at a 1:1 ratio to make a cell-DMSO mixture. For example, 1 mL of FBS, 3 mL of DMEM, and 1 mL of DMSO were mixed with 5 mL of the cell suspension to make a 10 mL cell-DMSO mixture. Next, the cell-DMSO mixture was quickly dispensed in cryovials (1 mL/vial) and the cryovials were placed in a freezing container and stored at −20 °C for 30 min. The freezing container was transferred into a −80 °C freezer overnight and then into a liquid nitrogen storage tank.

### 2.5. Testis Organoid Culture System

The isolated testis cells were first cultured in U-bottom 96-well plates (Cat. No. 4520; Corning, Kennebunk, ME, USA) placed in an incubator with 5% CO_2_ at 37 °C for 24 h to allow cells to form cell spheroids. A stable culture environment was provided, and the handling of culture plates was minimized during cell spheroid formation. The cell spheroids were then cultured on small blocks (1 × 1 × 0.5 cm) of 1.5% agarose gel bedding (Cat. No. 97062-250; VWR Amresco, Solon, OH, USA) immersed in DMEM supplemented with 10% KSR (Figure 1). Notably, the agarose gel base was presoaked in the media for 24 h to replace the water in the agarose gel with the media. The media were changed before placing the cell spheroids on the agarose gel base for further air–liquid interface culture. The spheroids were maintained at an air–liquid interface in an incubator with 5% CO_2_ at 37 °C to allow simultaneous gaseous and nutrients passage. The cell spheroids were further cultured for 4 weeks in the same air–liquid interface system to form the testis organoids. Approximately 80% of the media was changed every other day, and fresh media was added until reaching the same level as the top surface of the agarose gel base. It is worth noting that the organoids can be slightly immersed in the media but should not be completely covered by the culture media. Excessive culture media can cause the tissue to drift off the agarose gel, and potentially affect the gaseous exchange of the organoids. A drop of media was dispensed on the organoids to avoid the organoid from drying out during the procedure of changing media. The samples were collected weekly for histological analysis. We investigated different cell densities including 1.0 × 10^6^ vs. 0.8 × 10^6^ vs. 0.6 × 10^6^ testis cells/organoid to determine the optimal cell density for efficient tubulogenesis in organoids. To further optimize the culture condition to support testis organoids, we also tested several media supplementations (10% KSR, 10% FBS, 10% KSR + 5% FBS, or 5% KSR + 10% FBS) for efficient tubulogenesis and germ cell maintenance in the organoids.

### 2.6. Histological Analysis

Samples were fixed in Bouin’s solution for 4 h, then rinsed with and stored in 70% ethanol. The samples were processed using standard histological preparations, embedded in paraffin, and sectioned at 5 μm thickness. The sections were deparaffinized and stained with hematoxylin and eosin (H&E) or used for immunohistochemistry (IHC). The H&E staining was performed using previously described protocols [33]. Histological analysis of slides was performed using a light microscope equipped with digital photomicrography (Northern Eclipse Image Analysis software version 7.0, Empix Imaging, Mississauga, ON, Canada). The tubular relative area (% of tubular area in the cross-section compared with the total cross-sectional area of an organoid) was used as a quantitative parameter to compare the tubulogenic efficiency in different experimental groups. The relative germ cell number (% of germ cells compared with total cells on a cross-section of an organoid) was used as a quantitative measure of germ cell maintenance. The quantification of cross-sectional areas and cell numbers were performed on histological micrographs using ImageJ software.

### 2.7. Immunohistochemistry (IHC) and Tissue-Specific Staining

We performed immunohistochemistry (IHC) and tissue-specific staining to identify major cell types, cell orientations, and structural components of the organoids. Primary antibodies included anti-UCHL1 antibody (1:900, Cat. No. ab8189; Abcam, Cambridge, MA, USA, to detect gonocytes and early germ cells), anti-GATA-4 antibody (1:200, Cat. No. sc-1237; Santa Cruz Biotechnology, Santa Cruz, CA, USA, to detect Sertoli cells), anti-CYP17A1 antibody (1:50, Cat. No. sc-374244; Santa Cruz Biotechnology, to detect Leydig cells), anti-α-SMA (1:100, Mouse anti-α-SMA clone ASM-1, Cat. No. PA0943; Leica Biosystems Inc, Buffalo Grove, IL, USA, to detect PTMCs and vascular smooth muscle cells), and anti-vWF (1:1000, Rabbit anti-human vWF, Cat. No. IR527; Agilent Technologies Canada Inc., Mississauga, ON, Canada, to detect vascular endothelial cells). Secondary antibodies included the HRP-labeled anti-mouse/rabbit secondary antibody (universal anti-mouse/rabbit Ig, Cat. No. MP-7500; Vector Laboratories, Burlingame, CA, USA) and anti-goat secondary antibody (1:100, Cat. No. ab97100; Abcam). Detailed information for the antibodies has been listed in Supplementary Table S1. Each primary antibody was tested on testis tissue from 1-week-old piglets as a positive anatomical control for IHC. All secondary antibodies were also tested on testis tissues from 1-week-old piglets as negative controls to ensure that the incubation of the second antibody alone (without primary antibody) would not cause non-specific binding (Supplementary Figure S1). IHC for UCHL1, GATA4, and CYP17A1 were performed manually. Briefly, paraffin-embedded tissue sections were deparaffinized with xylene and rehydrated with graded ethanol. Heat induced antigen retrieval was performed at 98 °C in citrate buffer (pH 6.3, Cat. No. H-3300; Vector Laboratories) and Tris-EDTA buffer (pH 9.2–9.4, 1.21 g/L of Tris and 0.37 g/L of EDTA in distilled water) for 30 min, respectively; the tissue sections were then washed in DPBS three times. Next, endogenous peroxidase was inactivated by 0.3% hydrogen peroxide in distilled water for 15 min at 37 °C and the tissue sections were washed three times with DPBS. The tissue sections were then incubated overnight at 4 °C with the primary antibody in 2.5% horse serum (blocking agent, Cat. No. MP-7500; Vector Laboratories). After the primary antibody binding, the tissue sections were rinsed three times with DPBS and incubated with HRP-labeled anti-mouse/rabbit secondary antibody or anti-goat secondary antibody for 1 h at room temperature. The tissue sections were then incubated with DAB chromogen (Cat. No. SK-4105; Vector Laboratories) for 3 min and counter-stained with hematoxylin for 5 min. Sections were dehydrated with graded ethanol and xylene and sealed with mounting media and cover slips. On the other hand, IHC for α-SMA and vWF were performed using an automated staining platform. Briefly, heat-induced epitope retrieval was performed, and the primary antibodies were applied for 30 min. This was followed by detection using an HRP-labelled polymer reagent (EnVision+ System—HRP Labelled Polymer, Cat. No. K4003; Agilent Technologies), and the staining was visualized using 3,3′-diaminobenzidine tetrahydrochloride (DAB)^h^ as the chromogen (Dako Liquid DAB+ Substrate Chromogen System, Cat. No. GV823; Agilent Technologies). Masson’s trichrome (MT) and periodic Schiff-methenamine (PASM) staining were also performed using an automated staining platform. Histological analysis of slides was performed using a light microscope equipped with digital photomicrography (Northern Eclipse Image Analysis).

### 2.8. Transmission Electron Microscopy (TEM)

Transmission electron microscopy (TEM) was performed to examine the ultrastructure of the organoids. The fixation, embedding, and sectioning of samples for TEM were performed using our previously reported protocols [34]. Briefly, the tissue samples were first fixed in ice-cold 2% glutaraldehyde in 0.1 M sodium cacodylate buffer (4 °C, pH 7.2) for 4 h. The fixed tissue samples were then washed by immersion in 0.1 M sodium cacodylate buffer for 10 min and stored in fresh 0.1 M sodium cacodylate buffer at 4 °C. Post-fixation was performed by immersion in 1% osmium tetroxide for 1 h at room temperature. The tissue samples were then washed by distilled water and dehydrated with graded ethanol. Next, tissue samples were immersed in LR White resin mix diluted with pure ethanol in 1:1 and 2:1 ratios for 1 h, respectively, and finally in pure LR White resin mix for 2 h. The samples were then polymerized at 65 °C overnight and sectioned at 100 nm using a Leica Ultracut UCT. The sectioned samples were observed under a transmission electron microscope (HT7700; Hitachi, Tokyo, Japan) at an accelerating voltage of 80 kV.

### 2.9. Luteinizing Hormone (LH) Induction and Testosterone Measurements

In intact testis, testosterone secretion by Leydig cells is induced by luteinizing hormone (LH). To investigate the release of testosterone by the organoids’ Leydig cells in the culture media and their LH-responsiveness, we supplemented the organoid culture media with LH (100 ng/mL, Lutropin-V; Bio-niche Animal Health, Quebec City, QC, Canada) starting at day six of culture until the end of experiments (4 weeks). The organoid culture media were collected every other day for the measurements of testosterone levels (ng/mL) using testosterone ELISA kits (Cat. No. 582701; Cayman, Ann Arbor, MI, USA). Samples were also collected from organoid culture media that did not receive LH supplementation for comparison.

### 2.10. Statistical Analyses

All quantitative data were obtained from a minimum of three independent experiments using separately isolated testis cells. Statistical analyses were performed using SPSS (Version 25.0, IBM SPSS Statistics for Windows, Armonk, NY, USA). All data are expressed as means ± SEM. *p* < 0.05 is considered significant. The data from tubular relative areas and germ cell ratios were analyzed using two-way analysis of variance (ANOVA) with Tukey’s post-hoc test. Percentages were transformed (using Arcsin function) prior to ANOVA. The data from testosterone levels were analyzed by Welch’s ANOVA with Games–Howell post-hoc test. The data were log transformed prior to ANOVA for normalization.

## 3. Results

### 3.1. Both Fresh and Cryopreserved Testis Cells can Form Organoids

**Figure 2:**
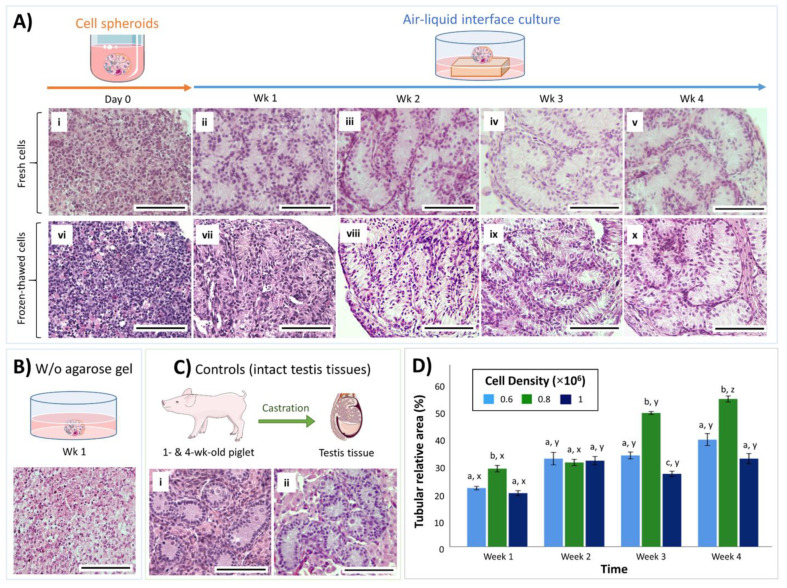
Representative histological micrographs and graphical data summary of tubulogenesis in testis organoids over the period of culture. (**A**) Histological micrographs of cell spheroids and testis organoids formed after culturing of fresh or frozen-thawed piglet testis cells. (**A**(**i**)) Cell spheroids on day 0 comprised of randomly distributed testis cells without evidence of tubulogenesis. (**B**) Cell spheroids submerged in the culture media (without the support of an agarose base) did not form testis tubular structures after 1 week of culture, and instead most cells underwent apoptosis or necrosis. (**A**(**ii**–**v**)) Cell spheroids cultured in the air–liquid interface system showed initial formation of testis tubules at week 1 (**A**(**ii**)), were further developed at week 2 (**A**(**iii**)), were maintained to week 3 (**A**(**iv**)), and week 4 (**A**(**v**)). (**A**(**vi**–**x**)) Frozen-thawed testis cells were also capable of self-assembling to form testis tubular structures that were similar to those of fresh cells. (**C**) Control tissues included intact testis tissues from 1-week-old (**C**(**i**)) and 4-week-old (**C**(**ii**)) piglets. W/o: without; Scale bars: 100 µm. (**D**) Tubular relative area (% of cross-sectional tubular area compared with the total area of the histology section) of testis organoids with different cell densities over time. Data are presented as mean ± SEM. ^abc^ Data with different letters differ significantly among cell density groups (*p* < 0.05). ^xyz^ Data with different letters differ significantly over time (*p* < 0.05). *n* = 3 replications.

When the isolated neonatal porcine testis cells were deposited in low-attachment U-bottom wells and allowed to settle for 24 h, they formed a coherent cell spheroid in each well (Figure 1A). Histological examination of the cell spheroids prior to culturing (day 0) showed that testis cells were closely packed; however, no tubular reconstruction was observable (Figure 2A(i)). The cell spheroids were then cultured for up to 4 weeks using either an air–liquid interface (on top of an agarose gel block base, Figure 2A(ii–v)) or submerged in the media (directly at the bottom of the culture plate and without the support of an agarose base, Figure 2B). Weekly samples were examined histologically and compared with intact testis tissues from 1-, and 4-week-old piglets (Figure 2C(i,ii)). Cell spheroids cultured directly in the media (without the support of agarose base) did not form any tubular structures after 1 week of culture, and most cells contained pyknotic nuclei or showed necrotic changes (Figure 2B). On the other hand, as early as 1 week after culture, formation of testis tubular structures resembling intact testis cords was observed in cell spheroids cultured in the air–liquid interface system (on an agarose base) (Figure 2A(ii)). Moreover, these testis tubular structures could be maintained for at least 4 weeks (the end of experiments) in the air–liquid interface system (Figure 2A(iii–v)).

To test whether organoids can also be generated from cryopreserved testis cells, frozen-thawed testis cells from 1-week-old piglets were used to form organoids using the same culture system. Similar to fresh cells, the organoids derived from cryopreserved testis cells also formed tubular structures as early as 1 week after culture (Figure 2A(vi,vii)) and could be maintained for at least 4 weeks (Figure 2A(viii–x)).

### 3.2. Cell Density Affects In Vitro Tubulogenesis of Organoids

To examine the effect of cell density on in vitro tubulogenesis of organoids, we quantified the relative area of tubular cross-sections in organoids resulting from different cell densities (1.0 × 10^6^*,* 0.8 × 10^6^, or 0.6 × 10^6^ testis cells/organoid) (Figure 2D). The tubular relative area in the 0.8 × 10^6^ cell density group was greater than other groups at 1, 3, and 4 weeks after culture (*p* < 0.05). A significant increase in the tubular relative area was observed at least at one time point for each group over the length of culture. Such an increase was observed at week 2 for both 1.0 × 10^6^ and 0.6 × 10^6^ cell density groups, whereas it was observed at weeks 3 and 4 for the 0.8 × 10^6^ group (*p* < 0.05).

### 3.3. Media Supplementation Affects In Vitro Tubulogenesis and Germ Cell Ratios in Organoids

**Figure 3:**
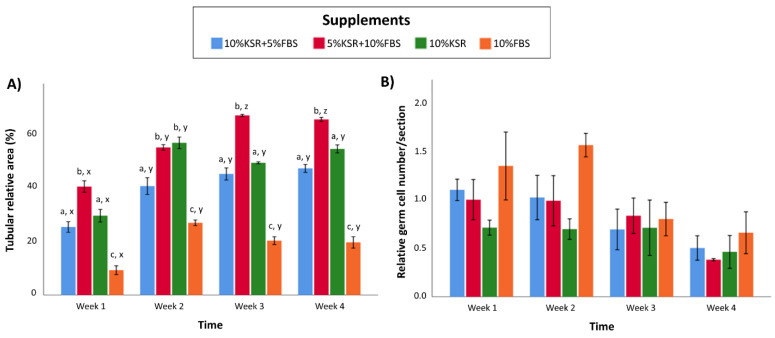
(**A**) Tubular relative area (% of cross-sectional tubular area compared with the total area of the histology section) and (**B**) relative germ cell numbers (% of germ cells to total number of cells in a histology cross-section) of testis organoids from different media supplementation groups over time. Data are presented as mean ± SEM. ^abc^ Data with different letters differ significantly among supplement groups (*p* < 0.05). ^xyz^ Data with different letters differ significantly over time (*p* < 0.05). *n* = 3 replications.

To examine the effects of media supplementation on in vitro tubulogenesis of the organoids, the relative area of tubular cross-sections was quantified over time in different supplementation groups (10% KSR, 10% FBS, 10% KSR + 5% FBS, 5% KSR + 10% FBS) (Figure 3A). The tubular relative area was greater in the 5% KSR + 10% FBS group than other groups at week 1, 3, and 4 (*p* < 0.05). In comparison, the tubular relative area of the 10% FBS group was lower than other groups, regardless of the time of culture (*p* < 0.05). A significant increase in the tubular relative area was observed at least at one time point during the culture for every group. Such an increase was observed at week 2 of culture in all groups, and at week 3 for the 5% KSR + 10% FBS group (*p* < 0.05). In this media, the tubular diameters measured at 1 and 4 weeks of culture were 47 ± 1 μm (*n* = 3) and 54 ± 0.9 μm (*n* = 3), respectively.

To examine the effects of media supplementation on germ cell numbers in organoids, the relative number of germ cells in the organoids were quantified over time of culture (Figure 3B). Surprisingly, the relative number of germ cells did not significantly differ among supplementation groups (*p* > 0.05). Since the total number of cells was relatively lower in the 10% FBS group than other groups, the relative germ cell numbers appeared to be numerically (but not significantly) higher than other groups at weeks 1, 2, and 4. Moreover, although a slight numeric decrease in the number of germ cells was observed for all groups over time, no statistical differences were detected (*p* > 0.05).

### 3.4. Cell Types and Structural Components of Organoids Correspond with Intact Tissue

**Figure 4:**
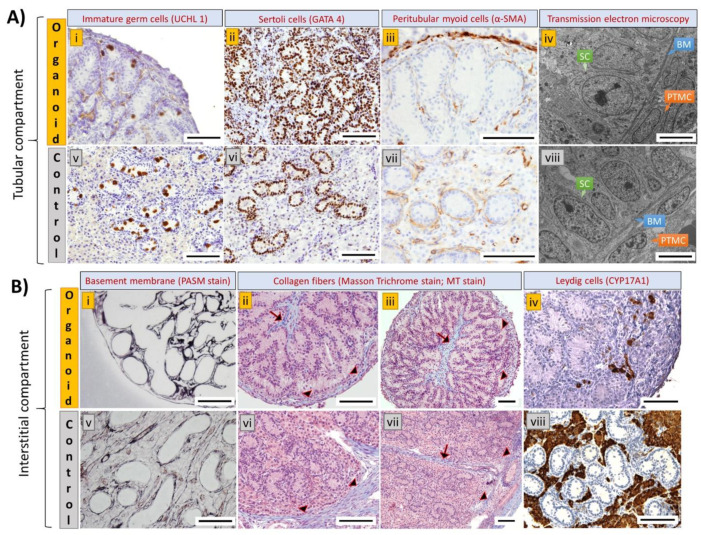
Immunohistochemistry, tissue-specific staining, and transmission electron microscopy (TEM) to detect tubular and interstitial compartments in testis organoids and intact testis tissues from 1-week-old piglets (positive anatomical controls). (**A**) Imaging of the tubular compartment in organoids (upper row) and intact testis tissue (lower row). Immature germ cells (**A**(**i**)), also known as gonocytes, were detected using UCHL1 and were observed within the tubular structures constructed by Sertoli cells (**A**(**ii**)) detected by GATA4, and peritubular myoid cells (**A**(**iii**)) detected by α-SMA. (**A**(**iv**)) A testis epithelium-like structure constructed by Sertoli cells (SCs), basement membrane (BM), and peritubular myoid cells (PTMCs) was observed in testis organoids as early as 1 week of culture. (**A**(**v**–**viii**)) Imaging of intact testis tissues (controls) with the corresponding cell types and structures. (**B**) Imaging of the interstitial compartment in organoids (upper row) and intact testis tissue (lower row). (**B**(**i**)) Peritubular basement membrane (black) was detected using periodic Schiff-methenamine (PASM) staining. (**B**(**ii**,**iii**)) Inter-tubular interstitium (arrows) and peripheral capsule (arrow heads) constructed by collagen fibers (blue) were detected in the organoids using Masson’s trichrome (MT). Also, Leydig cells (**B**(**iv**)) were detected by CYP17A and were observed in the inter-tubular interstitium. (**B**(**v**–**viii**)) Imaging of intact testis tissue (controls) with the corresponding cell types and structures. Scale bars: 100 µm (**A**(**i**–**iii**), **A**(**v**–**vii**), **B**(**i**–**viii**); 5 µm (**A**(**iv**,**viii**)).

**Figure 5:**
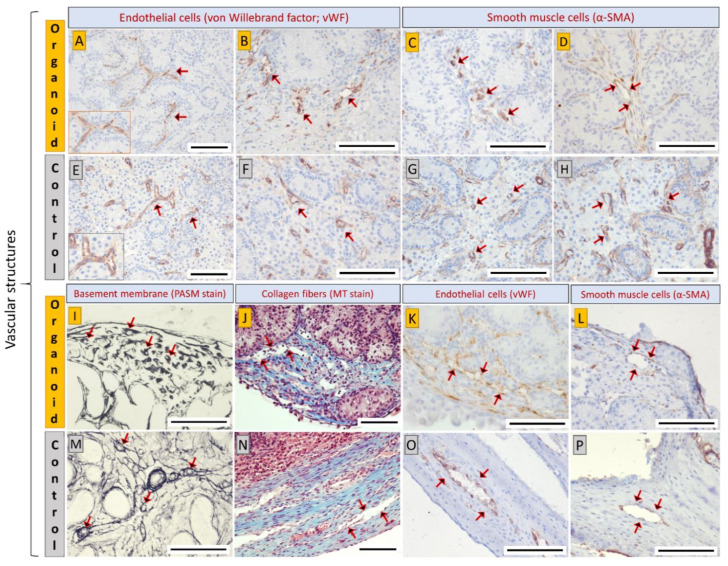
Tissue-specific staining and immunohistochemistry for endothelial (vWF) and smooth muscle cells (α-SMA) to detect vascular structures in the testis organoids and intact testis tissue from 1-week-old piglets (positive anatomical controls). (**A**,**B**) Microvascular structures were detected in the inter-tubular interstitium of the organoids, and these structures were constructed by vascular endothelial cells. (Inset in A) Branching of the microvascular structures was sporadically observed in the interstitium. (**C**,**D**,**I**) Microvascular structures contained smooth muscle cells (**C**,**D**) and perivascular basement membrane (**I**). (**J**–**L**) Sinusoid-like structures were observed in the peripheral capsule of the organoids, and these structures were also constructed by vascular endothelial cells (**K**) and smooth muscle cells (**L**). (**E**–**H,M**–**P**) Imaging of intact testis tissues with the corresponding staining and immunohistochemistry for vascular structures and cell types. PASM stain: Periodic Schiff-methenamine staining; MT stain: Masson’s trichrome staining; Scale bars: 100 µm.

To identify the cell types and structural components of the organoids, we performed IHC and tissue-specific staining. The organoids displayed a compartmentalized architecture resembling the tubular and interstitial compartments in the native testis tissue (Figure 4A,B). Notably, UCHL1-positive gonocytes (Figure 4A(i)) were located in the tubular structures reconstructed by GATA4-positive Sertoli cells (Figure 4A(ii)) and α-SMA-positive PTMCs (Figure 4A(iii)). However, compared with the innate testis tissue, the overall number of gonocytes was low and in a few instances, they were found outside of the tubular structures. Upon TEM imaging, testis epithelium-like structures comprising Sertoli cells, basement membrane, and PTMCs resembling native testis epithelium were observed in the organoids (Figure 4A(iv)). Based on PASM and MT staining, peritubular basement membrane was observed to encircle the tubular structures (Figure 4B(i)), while the inter-tubular space was occupied with collagen fibers creating an interstitium compartment in the organoids (Figure 4B(ii,iii)). A testis capsule-like structure constructed by collagen fibers was also observed at the periphery of the organoids (Figure 4B(ii,iii)). Furthermore, CYP17A1-positive Leydig cells were observed in the inter-tubular interstitium and located mainly at the periphery of the organoids (Figure 4B(iv)). Surprisingly, microvascular and sinusoid-like structures were observed within the inter-tubular interstitium and in the peripheral capsule of the organoids (Figure 5A–D,I–L), which resemble the vascular structures of the native testis tissue (Figure 5E–H,M–P). The interstitial microvasculature was structurally similar to immature vasculature (nascent vessels) constructed by a single layer of vWF-positive vascular endothelial cells (Figure 5A,B) [35]. Branching of microvasculature was occasionally observed in the interstitium (inset in Figure 5A), which resembled the microvascular network of the native testis tissue (inset in Figure 5E). Furthermore, α-SMA-positive smooth muscle cells (Figure 5C,D) and perivascular basement membrane (Figure 5I) were also detected in some vascular structures. Lumen was found in some of these vascular structures, but not always visible due to the absence of blood cells. On the other hand, sinusoid-like structures observed at the peripheral capsule were also constructed by a single layer of endothelial cells (Figure 5J,K), and occasionally lined with smooth muscle cells (Figure 5L).

### 3.5. Leydig Cells in Organoids Secrete Testosterone in Response to LH Stimulation

**Figure 6:**
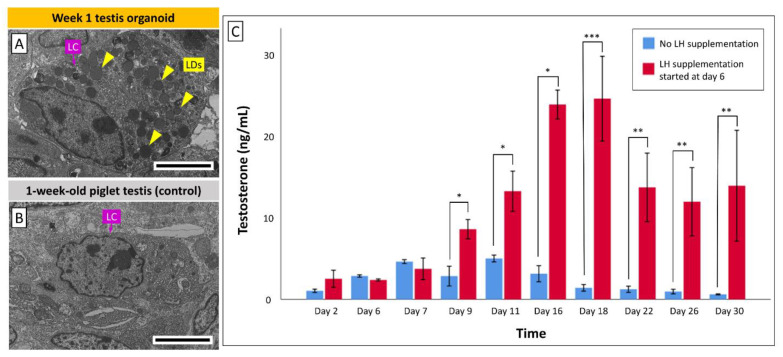
Transmission electron microscopy (TEM) and testosterone measurements to study the androgen synthesis and luteinizing hormone (LH)-responsiveness of Leydig cells (LCs) in testis organoids. (**A**) Lipid droplets (LDs) were observed in an LC of organoid as early as 1 week of culture, indicating initial androgen synthesis and functional maturation of LCs in the organoids. (**B**) An LC in intact testis tissue from a 1-week-old piglet (control) does not contain any LDs. Scale bars: 5 µm. (**C**) Testosterone levels (ng/mL) in the organoid culture media were measured over time via ELISA. Red columns represent LH supplemented groups: LH supplementation in the organoid culture media started at day 6 of culture and continued until the end of experiments (day 30). Blue columns represent control groups without LH supplementation. The higher levels of testosterone during days 9 to 30 of culture suggest that Leydig cells in the organoids can be induced by LH to release more testosterone. Data are presented as mean ± SEM. Data with asterisks differ significantly among groups (*p*: * <0.05, ** <0.01, *** <0.005). *n* = 3 replications.

Based on TEM imaging, numerous lipid droplets were observed within the Leydig cells in week 1 organoids (Figure 6A), while Leydig cells in the control testis tissue from 1-week-old piglets contained no or only a few lipid droplets (Figure 6B). This suggested the initiation of in vitro maturation of Leydig cells in the organoids (Figure 6A). To determine the androgen synthesis ability and potential LH-responsiveness of testis organoids, we measured testosterone levels (ng/mL) in the organoid culture media over the course of culture (Figure 6C). LH was added to the culture media of a group of organoids starting at day 6 of culture, while the negative control group of organoids did not receive such an LH supplementation. During days 2 to 7 of culture, no statistical differences in testosterone levels were observed between the control and LH supplemented groups (*p* > 0.05). However, starting on day 9 of culture and through the end of this experiment (day 30), testosterone levels in the LH-supplemented group were significantly higher than the control group (*p* < 0.05), suggesting that Leydig cells in the organoids can be induced by LH to release more testosterone.

## 4. Discussion

We have previously reported that de novo testis tubulogenesis can be induced by the in vivo implantation of primary testis cell aggregates, derived from neonatal piglets, under the skin of immunodeficient mice to form a compartmentalized testis tissue [36,37]. In the present study, we demonstrated that neonatal porcine testis cells also possess an in vitro tubulogenesis ability to form a compartmentalized testis organoid, similar to the reported mouse testis organoids [15,38]. Among various testis organoid culture systems, cell spheroid culture method has been proven effective in initiating the reconstruction of a compartmentalized testis organoid in rodent models [15,38]. This methodology allows cells to be freely suspended in the media instead of being attached to the walls of the culture well. With the gravitational force, dissociated cells are condensed at the center of the well, which maximizes cell-to-cell contact and facilitates the formation of a 3D cell spheroid. A key factor in the success of the present culture system was the air–liquid interface culture which improves nutrient perfusion and gaseous exchange in the organoids. This was highlighted when we observed that cell spheroids cultured while submerged in the media (without the support of an agarose base) do not form any tubular structures and most cells undergo apoptosis or necrosis. This was also the case with murine testis organoids when using a similar culture method [38]. The air–liquid interface culture system has been widely used in testis tissue cultures to minimize hypoxia-induced cell death [39]. Other systems such as the microfluidic and rotation culture systems have also been developed to continuously provide fresh media to the tissue and increase the surface area for nutrient/oxygen diffusion [40,41].

In the present study, we also showed that frozen-thawed neonatal porcine testis cells can generate testis organoids similar to organoids derived from fresh testis cells. Similar results were also reported by Sakib et al. (2019b) using a microwell culture system [20]. This is an important observation since it expands the feasibility of using cryopreserved testis cells for building testis organoids [2,42,43] in situations where using fresh cells is not practical or desired.

The initial number of cells used to form a testis organoid (i.e., cell density) has varied among different studies. In general, a higher cell density is expected to promote intimate cell-to-cell interactions, which should improve de novo testis organogenesis. However, high cell densities also increase the size of organoids hence causing poor perfusion of oxygen and nutrients to the center of the organoid, eventually leading to central necrosis. On the other hand, while lower cell density is beneficial in improving nutrient/oxygen perfusion in the organoids [44], organoid formation may be hindered due to insufficient number of initial testis cells [20]. In scaffold-free cell spheroid culture systems, cell densities ranging from 1.0 × 10^3^ to 2.0 × 10^6^ cells per organoid have been reported [15,20,38]. In our preliminary experiments (data not shown), we also tested using 2.0 × 10^6^ cells per organoid which caused central necrosis in the resultant organoids, while using 0.5 × 10^6^ cells/organoid resulted in delayed or impaired tubulogenesis, and the organoids were too small to be handled for histological processing. Therefore, mid-range cell densities including 0.6 × 10^6^, 0.8 × 10^6^, and 1.0 × 10^6^ cells/organoid were investigated further for their organoid formation and tubulogenic efficiency. In the present study, we evaluated tubulogenic efficiency by quantifying the relative area occupied by viable tubular cross-sections within a testis organoid. Therefore, a high tubular relative area indicates superior tubular reconstruction which was provided by 0.8 × 10^6^ testis cells per organoid as an optimal cell density to generate a porcine testis organoid.

Furthermore, there has been no consensus on the most suitable culture media supplementation for formation of testis organoids [2]. For serum-based culture media, FBS or KSR have been commonly used in organoid culture systems. FBS is abstracted from serum of fetal calves and hence contains various growth factors and biological substances with diverse effects on cells especially on their growth and development. These characteristics make FBS a common serum supplement for in vitro cell culture. The addition of 10% FBS in a 2D Sertoli cell line culture was reported to induce the formation of testis tubular structures [45], stimulate PTMC proliferation and support de novo testis tubulogenesis in vitro and in vivo [46,47]. However, the unidentified components in FBS as well as inconsistent quality due to the supplier and batch variability make it challenging for researchers to study the effects of individual components or their key mechanisms in inducing de novo testis tubulogenesis. Also, since the reconstruction of testis tubular structures has also been reported in a serum-free Matrigel-based 3D culture system [47], the key inducers in both FBS and Matrigel that trigger de novo testis tubulogenesis remain unknown. In comparison, KSR has defined components, consistent quality between batches, and hence is commonly used in embryonic stem cell cultures to avoid unwanted cell differentiations [48]. Sato et al. (2011) were first to show that KSR as a supplement can play a crucial role for the in vitro production of fertilization-competent murine spermatozoa [48]. Later, Yokonishi et al. (2013) used KSR supplementation in murine testis organoid system, which resulted in the formation of a compartmentalized testis organoid with tubular structures and partial spermatogenic differentiation [38]. Most testis organoid systems have used KSR as a media supplement to maintain spermatogonial proliferation in organoids [1,17], or to support spermatogenic differentiation from spermatogonia up to spermatocytes [49,50,51], spermatids [52], or even elongated spermatids [53]. Zhang et al. (2014) also showed that de novo testis tubulogenesis and the formation of compartmentalized seminiferous tubule-like structures only occurred in KSR supplemented groups [49]. A combined supplementation of KSR and FBS was reported to improve bovine SSC colonization in 2D cell cultures [54]. In the present study, we therefore investigated the effects of media supplementation by providing a side-by-side comparison of FBS, KSR, and their combinations to test the potential beneficial effects on de novo tubulogenesis. We evaluated the tubular relative area of organoids resulting from 10% KSR, 10% FBS, or combined supplementation of 10% KSR + 5% FBS or 5% KSR + 10% FBS. Our results indicated that compared with other groups, the combined supplementation of 5% KSR + 10% FBS improved de novo tubulogenesis of organoids, while the 10% FBS (only) group had the lowest tubular relative area and organoid sizes. This observation coincided with the relatively disorganized cellular arrangements and necrosis in the FBS-only supplemented organoids (Supplementary Figure S2A). Interestingly, our follow up results using a 2D cell culture showed that somatic cells (e.g., PTMCs, Sertoli cells, and fibroblasts) proliferate more rapidly in the FBS-only than in KSR-only supplemented media (Supplementary Figure S2B). Therefore, it appears that FBS promotes the proliferation of somatic testis cells which would theoretically better support the formation of testis cord-like structures in vitro [2,45]. However, given that all organoids started off with the same number of cells, the considerably lower tubular formation and organoid sizes in the FBS-only supplemented group can be explained by the excessive proliferation of somatic cells leading to lowered gaseous/nutrient exchange causing cell death. The fact that the tubular relative area of the 5% KSR + 10% FBS group was higher than both 10% KSR + 5% FBS and 5% KSR groups may also support this conclusion. These observations indicate that a combination of the two supplements at the given ratio (5% KSR + 10% FBS) is the optimal media supplementation for tubular reconstruction and maintaining the viability of porcine testis organoids. Next, to determine the effects of media supplementation on germ cell maintenance in organoids, the relative number of germ cells in different supplementation groups was quantified. Our findings indicated that changing supplements did not affect the relative number of germ cells in the testis organoids. Also, no significant decrease in the number of germ cells was observed in the organoids over the 4-week duration of culture. It is worth noting that the germ cell ratios appeared numerically (but not significantly) higher in the FBS-only group, likely because organoids in this group had a smaller size and lower total cell numbers, which would inversely increase the relative germ cell numbers (Supplementary Figure S2C).

Next, IHC and tissue-specific staining were performed to determine the cell types and structural components of the newly established testis organoids. Our findings showed that the testis organoids consisted of tubular and interstitial compartments resembling native testis tissue. Namely, testis tubular compartments were formed by Sertoli cells, PTMCs, and the peritubular basement membrane and gonocytes were almost exclusively observed within these tubules. Also, reconstruction of the inter-tubular interstitial compartments containing Leydig cells and collagen fibers was observed in testis organoids. As described earlier, native testis tissue is comprised of both interstitial and tubular compartments; each compartment contains mixed cell populations and distinctive spatial orientation. The cellular and acellular components in both compartments play a crucial role in the SSC niche to regulate SSC proliferation and/or differentiation [55]. Therefore, the presence of major testis cell types and biomimetic cell polarity are keys to fabricating a functional testis organoid that recapitulates the SSC niche [56]. Our organoid system has therefore fulfilled the three main criteria for organoid formation, including (a) testis cell reassembly, (b) the compartmentalized architectures, and (c) the inclusion of major testis cell types. Since not all testis organoid studies reported have performed a complete analysis according to the above criteria for organoid formation, it is difficult to fully compare all testis organoid studies [3]. For example, some studies did not perform immunostaining of Leydig cells and PTMCs, leading to incomplete interpretation of the overall architecture, cell types, and spatial orientation in their systems [19,38,57,58,59]. To our knowledge, only three testis organoid studies have thus far achieved all the criteria for forming organoids with biomimetic, compartmentalized, and complete testis tubular/interstitial structures comprising at least three major testis cell types [15,38,49].

Yokonishi et al. (2013) observed irregular architecture of the reconstructed testis tubular structures in mouse testis organoids; the tubular structures had uneven diameter and a maze-like configuration [38]. In addition, fewer Leydig and germ cells were observed in their organoids than in normal tissue [38]. França et al. (2000) reported that the relative germ cell numbers in intact testes (% of germ cells compared with the total number of Sertoli, Leydig, and germ cells per testis) were ~4.8% and ~2.8% in 1-day-old and 1-month-old piglets, respectively [60]. In comparison, the relative numbers of germ cells in our organoids were ~0.5% to 1.5%; lower than those observed in intact testis tissues. Similar architecture and cellular components were also observed in other testis organoid studies [15,21]. The causes and consequences of deformed testis tubular structures and unbalanced cell proportions remain to be investigated. Nevertheless, the size and expansion pattern of testis tubular structures in our testis organoids show similarity with the developing testes in intact pigs. Firstly, the testis tubular diameters in intact testes from 1-day to 1-month-old pigs were reported to be 53 ± 0.2 and 57 ± 2 μm, respectively [60], which are in the same range as those in our organoids (47 to 54 μm) after 1 and 4 weeks of culture, respectively. Secondly, França et al. (2000) observed that the testis tubular structures occupied ~40–50% of the parenchyma in intact testes from 1-day to 2-month-old pigs, which subsequently increased to ~70% in 3-month-old pigs [60]. Such an increase in tubular relative area was also observed in our testis organoids. For example, the tubular relative area increased from ~40% to ~65% in the organoids after 3 weeks of culture in 5%KSR + 10%FBS. In addition, these in vitro-produced structures are comparable to the in vivo reconstructed testis tubular structures using ectopic testis cell implantation [36,37,47,61,62], indicating that we are now capable of replicating the required microenvironment necessary for complete formation of testis tubulogenesis in vitro. On the other hand, proper cell maturation in the organoids is crucial for spermatogenesis and testis development. The deformation of seminiferous tubule and the absence of Leydig cell maturation were reported to hinder spermatogenesis in *Desert hedgehog* (*Dhh*) null mice [63]. Also, the maturation of Sertoli cells, especially the formation of blood-testis barrier (BTB), plays a critical role in regulating spermatogenesis [64]. Therefore, the maturational state of testis cells in organoids should be determined in future studies.

One of the most exciting findings in the present study was the formation of vascular structures in the testis organoids. During vascular formation, single-layered endothelial-cell tubes are first generated as immature vasculature (nascent vessels). Subsequently, this immature vasculature specializes and develops into microvessels such as capillaries, arterioles, and venules. Capillaries are constructed by endothelial cells and pericytes, and surrounded by a basement membrane; in comparison, arterioles and venules have an additional coverage of smooth muscle cells that are responsible for vasoconstriction and vasodilation [35]. In our organoids, the vascular structures were constructed by a single layer of endothelial cells, occasionally lined with smooth muscle cells, and surrounded by perivascular basement membrane. This finding suggests that the vascular structures in the organoids were mostly immature nascent vessels, but some vascular structures were developing into relatively mature microvessels such as capillaries, arterioles, or venules. Together, a mixed population of vascular-like structures, including immature nascent vessels and developing microvessels, was found in the organoids. To date, the formation of vascular structures has not been reported in any of the testis organoid culture systems. The requirement or significance of a vascular network in an in vitro culture system is still unknown, especially given the absence of red blood cells. At the very least, this observation indicates that the presence of blood is not a prerequisite for formation of blood vessels. Nevertheless, the presence of vascular structures in organoids might provide a previously unavailable in vitro model to examine various hypotheses related to the SSC niche and embryonic testis organogenesis. For example, the SSC niche is thought to require contributions from the nearby blood vessels to function [65]. However, it has been challenging to examine this hypothesis in an in vitro testis organoid system where the vasculature was absent. It is also challenging to test this hypothesis using in vivo systems due to the interference of host-derived hormones and growth factors. Next, vasculature is also important for another elusive mechanism, namely, the formation of testis cords during embryonic testis organogenesis. A current theory proposes that migrating endothelial cells are in control of signaling the pattern for testis cords formation during this period [66,67]. In other words, testis cords follow the pattern of vasculogenesis and not vice-versa. Again, this theory has been difficult to be examined using the currently available in vitro or in vivo systems. Therefore, our organoid culture system provides a promising platform for novel studies on the role of blood vessels in the SSC niche and testis organogenesis, among other blood vessel formation mechanisms.

In vitro maturation of fetal Leydig cells was reported using an air–liquid interface culture system [68]. To our knowledge, in vitro maturation of Leydig cells in neonatal testis cell-derived organoids has not yet been reported. In the present study, the presence of numerous cytoplasmic lipid droplets in Leydig cells of the organoids was observed, indicating that a certain degree of functional cell maturation could be achieved in these cells after one week of culture. In addition, the LH-supplemented organoids secreted significantly higher amounts of testosterone than the control organoids, suggesting that the Leydig cells in the organoids possess endocrine functionality and LH responsiveness. Stable secretion of testosterone was also reported in other neonatal testis cell-derived organoids in response to human chorionic gonadotropin (hCG) using mouse [15] and pig models [21]. hCG possesses α- and β-subunits that display homologies with LH [69], and exerts LH-like action on Leydig cells to induce testosterone secretion [70]. Furthermore, combined supplementation of FSH and hCG has also been reported to induce in vitro differentiation of SSCs to elongated spermatids in soft agarose- and methylcellulose-based testis cell cultures [71]. Therefore, more studies are required to compare the testosterone secretion in groups supplemented with hCG or LH.

Moreover, testosterone is a crucial hormone in supporting testis development and spermatogenesis, especially spermatogenic differentiation during meiosis and spermiogenesis stages [72,73,74]; hence, testosterone has been frequently supplemented in testis cell culture [75,76,77,78]. However, based on the results of the present study, the necessity of supplementation of both testosterone and LH into the testis cell culture is uncertain because testosterone can be produced by Leydig cells in response to LH induction [15,21]. More studies are required to evaluate the degree of organoid formation and in vitro spermatogenesis in groups with or without testosterone supplementation. Next, dihydrotestosterone (DHT), a 5α-reduced metabolite of testosterone, is a more potent androgen that supports spermatogenesis and androgen-mediated events at puberty [79,80]. The exact mechanisms by which DHT applies a higher potency are unclear, but DHT binds more tightly to androgen receptors leading to amplification of downstream gene transcription at lower concentrations than testosterone [81]. The ELISA kit used in this study has only ~27% cross reactivity with DHT; but to our knowledge, no testis organoid study has specifically measured the DHT produced from the organoids. Hence, it will be interesting to investigate whether the organoids can secrete DHT.

## 5. Conclusions

We established a new testis organoid culture system using neonatal porcine testis cells by first forming cell spheroids in low attachment culture wells followed by long-term culture in an air–liquid interface system. Both fresh and cryopreserved testis cells were fully capable of forming testis organoids comprising testis cord-like structures, which could be maintained for at least 4 weeks. A testis cell density of 0.8 × 10^6^ cells/organoid and a combined supplementation of 5% KSR + 10% FBS in media were deemed optimal for improving tubulogenesis of the organoids. Furthermore, the number of germ cells (gonocytes) did not significantly decrease over the 4 weeks of culture in any of the media supplementation groups. Our testis organoids consist of tubular and interstitial compartments resembling the innate testis tissue. Importantly, the cell types and their orientations also closely correspond to the innate testis tissue, including the formation of testis tubular compartments by Sertoli cells, PTMCs, and peritubular basement membrane which also encompass germ cells. Formation of the inter-tubular interstitial compartments containing Leydig cells, collagen fibers, and most notably vascular structures was also observed in the organoids. Furthermore, the testis organoids have endocrine functionality of testosterone secretion and LH-responsiveness. Therefore, these findings provide a robust, accessible, and tunable model for a wide range of basic research and diagnostic applications. This includes exploring the contribution of individual cell types, the effects of various matrix materials, and potential consequences of known or unknown factors to gain insights into the testis development. As such, this testis organoid system can prove valuable in embryology and reproductive biology research, pharmaco-toxicology testing, and regenerative medicine.

## Data Availability

The visual and numeric data included in this article are available upon request to further support the conclusions.

## Supplementary Materials

The following are available online at https://www.mdpi.com/article/10.3390/cells10071696/s1. Table S1: Primary and secondary antibodies used for immunohistochemistry. Figure S1: Negative controls for secondary antibodies. Figure S2: Representative histological micrographs of testis organoids cultured in media supplemented with either fetal bovine serum (FBS) or a combined supplementation of FBS and knock-out serum replacement (KSR).
