# Supplementary material for: Generation of a Highly Biomimetic Organoid, Including Vasculature, Resembling the Native Immature Testis Tissue
Source: Cells. 2021 Jul 5;10(7):1696. doi: 10.3390/cells10071696 (PMC8305979; doi:10.3390/cells10071696)
Supplement: Supplementary file 1 [file cells-10-01696-s001.zip › cells-1259323-SI.pdf]

cells

MDPI

Supplementary materials

Table S1. Primary and secondary antibodies used for immunohistochemistry.

Antibody	Target cell type/primary antibody	Dilution	Distributor / Product Number	Ref. for antibody reactivity on pig tissue
**UCHL1**	Gonocytes and early germ cells	1:900	Abcam / ab8189	[1, 2]
**GATA-4**	Sertoli cells	1:200	Santa Cruz Biotechnology / sc-1237	[3, 4]
**CYP17A1**	Leydig cells	1:50	Santa Cruz Biotechnology / sc-374244	[5]
**α-SMA**	PTMCs and vascular smooth muscle cells	1:100	Leica Biosystems Inc / PA0943	[6]
**vWF**	Vascular endothelial cells	1:1000	Agilent Technologies Canada Inc / IR527	[7]
**HRP labelled anti-mouse/rabbit**	UCHL1 and CYP17A1 antibody	-	Vector Laboratories / MP-7500	N/A (secondary antibody)
**HRP labelled anti-goat**	GATA-4 antibody	1:100	Abcam / ab97100	
**EnVision+ System- HRP labelled polymer anti-mouse**	α-SMA antibody	-	Agilent Technologies / K4000	
**EnVision+ System- HRP labelled polymer anti-rabbit**	vWF antibody	-	Agilent Technologies / K4002	

References for table S1:

1. Zheng, Y.; Feng, T.; Zhang, P.; Lei, P.; Li, F.; Zeng, W. Establishment of cell lines with porcine spermatogonial stem cell properties. *Journal of animal science and biotechnology* **2020**, 11, 1-12, doi: 10.1186/s40104-020-00439-0.
2. Zhang, P.; Li, F.; Zhang, L.; Lei, P.; Zheng, Y.; Zeng, W. Stage-specific embryonic antigen 4 is a membrane marker for enrichment of porcine spermatogonial stem cells. *Andrology* **2020**, *8*, doi: 1923-1934. 10.1111/andr.12870.
3. Legacki, E.; Conley, A.J.; Nitta-Oda, B.J.; Berger, T. Porcine Sertoli cell proliferation after androgen receptor inactivation. *Biology of reproduction* **2015**, 92, 93, 91-97, doi: 10.1095/biolreprod.114.125716.
4. Wang, W.-Y.; Meng, L.-J.; Xu, Y.-J.; Gong, T.; Yang, Y. Effects of 4% paraformaldehyde and modified Davidson's fluid on the morphology and immunohistochemistry of Xiang pig testes. *Journal of Toxicologic Pathology* **2020**, 2019-0072, doi: 10.1293/tox.2019-0072.
5. Yu, S.; Zhang, P.; Dong, W.; Zeng, W.; Pan, C. Identification of stem leydig cells derived from pig testicular interstitium. *Stem cells international* **2017**, 2017, doi: 10.1155/2017/2740272.
6. Ueda, S.; Nunn, B.M.; Chauhan, R.; McDonald, K.; Kaplan, H.J.; O'Toole, M.G.; Tamiya, S. Sustained dasatinib treatment prevents early fibrotic changes following ocular trauma. *Graefe's Archive for Clinical and Experimental Ophthalmology* **2021**, 259, 1103-1111, doi: 10.1007/s00417-020-05037-4.
7. Eshmuminov, D.; Becker, D.; Borrego, L.B.; Hefti, M.; Schuler, M.J.; Hagedorn, C.; Muller, X.; Mueller, M.; Onder, C.; Graf, R. An integrated perfusion machine preserves injured human livers for 1 week. *Nature biotechnology* **2020**, 38, 189-198, doi: 10.1038/s41587-019-0374-x.

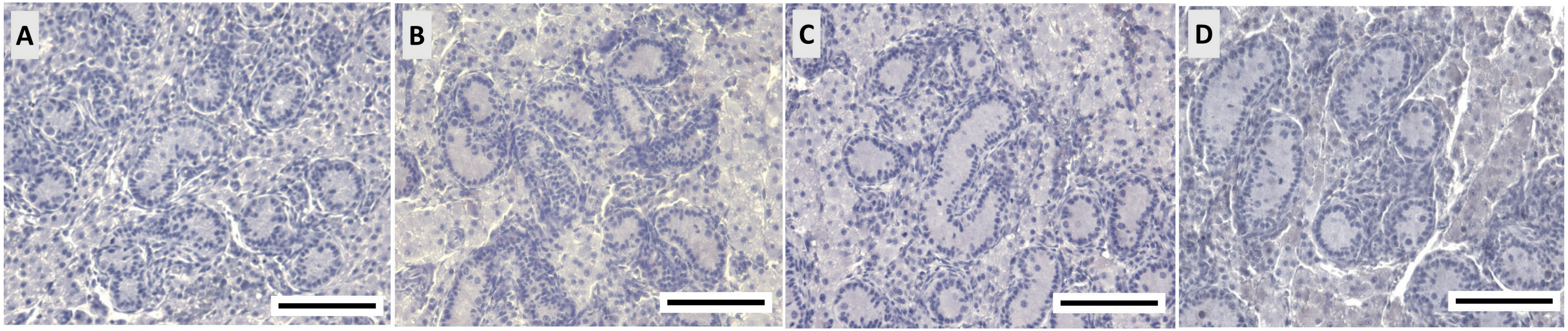

Figure S1. Negative controls for secondary antibodies. (**A-D**) Non-specific binding was not observed when secondary antibodies were used for immunostaining of 1-week-old piglet testis tissues without the addition of primary antibodies. (**A**) HRP labelled anti-mouse/rabbit; (**B**) HRP labelled anti-goat; (**C**) EnVision+ System- HRP labelled polymer anti-mouse; and (**D**) EnVision+ System- HRP labelled polymer anti-rabbit.

cells

MDPI

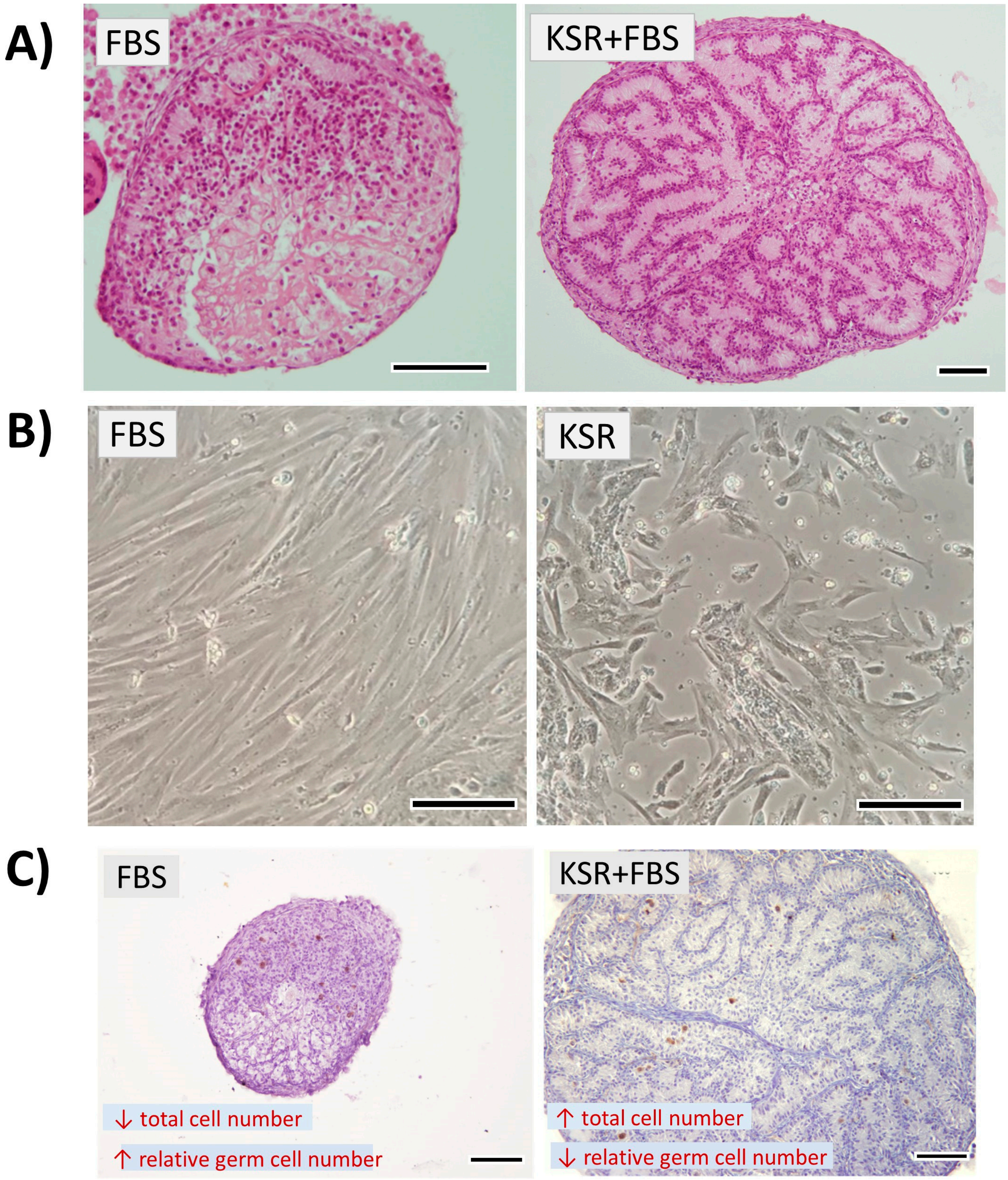

Figure S2. Representative histological micrographs of testis organoids cultured in media supplemented with either fetal bovine serum (FBS) or a combined supplementation of FBS and knock-out serum replacement (KSR). (**A**) A testis organoid from the FBS group (left) showing a lower tubular relative area and large areas of necrosis, whereas a testis organoid from the KSR+FBS group (right) showing a higher tubular relative area. (**B**) Two-dimensional (2D) culture of testis cells in media supplemented with either KSR or FBS. Rapid proliferation of somatic cells cultured in FBS-supplemented media (left) compared with those in KSR-supplemented media (right). (**C**) Representative histological micrographs of testis organoids in different media supplementation groups. A testis organoid from the FBS group showing a smaller size and lower total cell numbers, which inversely increased the relative number of germ cells (left), whereas organoids in the KSR+FBS groups

had a larger size and higher total cell numbers (right). Gonocytes (immature germ cells) were detected using UCHL1 immunostaining (brown).
